# Using novel porous metal pillars for tibial bone defects in primary total knee arthroplasty

**DOI:** 10.1186/s12891-023-06962-1

**Published:** 2023-10-20

**Authors:** Qiheng Tang, Shaoyi Guo, Wang Deng, Yixin Zhou

**Affiliations:** Department of Orthopedics, Beijing Jishuitan Hospital, Capital Medical University, The Fourth Clinical Medical College of Peking University, No. 31 Xinjiekou East Street, Xicheng District, Beijing, 100035 China

**Keywords:** Bone defect, Total knee arthroplasty, Porous metal pillar, Augmentation

## Abstract

**Background:**

The optimal method to treat tibial bone defects during primary total knee arthroplasty (TKA) is still unclear. A novel technique of porous metal pillar augmentation has been applied recently. This study aimed to assess the short-term outcomes of primary TKA with the use of novel porous metal pillars for tibial bone defects.

**Methods:**

A total of 24 cases (22 patients) of primary TKA between January 2019 and December 2020 using porous metal pillars for tibial bone defects were reviewed. Clinical results were evaluated using the Knee Society knee score (KSKS) and function score (KSFS), the Western Ontario and McMaster Universities Osteoarthritis Index (WOMAC), and range of motion (ROM). Hip-knee-ankle angle (HKAA), femorotibial angle (FTA), and radiolucent lines were assessed radiologically.

**Results:**

The median follow-up period was 36.0 months (interquartile range: 31–37 months). The KSKS, KSFS, WOMAC score, and ROM improved significantly at the final follow-up assessment compared with the preoperative evaluation. Both of the HKAA and FTA were corrected after surgery. Only one knee had a nonprogressive radiolucent line at the bone-cement interface. No radiolucent lines were detected around the pillar in any of the cases. There were no cases of prosthesis loosening and revision.

**Conclusions:**

The use of novel porous metal pillars yielded satisfactory clinical outcomes and reliable radiological evidence of fixation in this study with a minimum 2-year follow-up. Porous metal pillar augmentation can be considered as a valuable and easy-to-use method for the management of tibial bone defects in primary TKA.

## Background

 Tibial bone defects are frequently encountered during primary total knee arthroplasty (TKA). According to the classification of bone defects by Rand [1], the extent of bone loss may be subdivided into minimal defect with a depth of < 5 mm, moderate defect of 5–10 mm, extensive defect of ≥ 10 mm, and massive cavitary defect. Poorly treated bone defects can impact the stability of the tibial prothesis. In particular, the management of large bone defects poses a significant challenge for the surgeon.

Traditional methods to treat tibial bone defects include increased bone resection, bone cement filling, bone grafting, and metal augmentation. Increased tibial bone resection is indicated for minimal bone defects because the strength of the tibial bone decreases with more distal tibial resection [2, 3]. Bone cement with or without screws can be used to fill minimal and moderate bone defects [4, 5]. The disadvantage of bone cement filing is that it may not provide adequate support for the tibial component [6]. Although bone grafting allows for restoration of bone stock, this technique has several complications such as bone resorption, collapse, nonunion, and infection [7, 8]. Metal wedge or block augment is commonly used to address large bone loss [9, 10]. However, further bone resection is needed to fit the metal augment, which may reduce bone stock and make future revision surgery difficult.

Recently, a novel technique of porous metal pillar augmentation has been applied to treat bone defects in knee surgery with the theoretical advantages of immediate mechanical support and long-term stability [11, 12]. To the best of our knowledge, similar technique has not been reported previously. The purpose of the current study was to determine the short-term clinical and radiographic results with the use of porous metal pillars for tibial bone defects during primary TKA.

## Methods

This study was approved by the Institutional Review Board of our hospital. Between January 2019 and December 2020, a total of 24 patients who underwent primary TKA for osteoarthritis with the use of porous metal pillars for tibial bone defects were eligible for inclusion. The porous metal pillars were indicated to treat tibial bone defects when the bone defects were peripheral and uncontained, and the extent of bone defects were moderate and extensive according to the classification of bone defects by Rand [1]. Of these 24 patients, 2 patients without 2-year follow-up were excluded. Finally, the remaining 22 patients (24 knees) with a follow-up period of at least 2 years were enrolled in this study.

The mean age of the patients was 65.4 years (range, 57–77 years) at the time of surgery. There were 17 females and 5 males. The mean body mass index (BMI) was 26.8 kg/m^2^ (range, 20.0-35.6 kg/m^2^). The follow-up period was from 25 to 47 months and the median was 36.0 months (Table 1). The diagnosis was degenerative osteoarthritis in all patients. There were 22 varus knees and 2 valgus knees. The prostheses included Genesis II (Smith & Nephew, Memphis, TN, USA) in 21 knees, Legion (Smith & Nephew, Memphis, TN, USA) in 1 knee, and LCCK (Zimmer, Warsaw, IN, USA) in 2 knees. All prostheses were fixated with bone cement.

**Table 1:** Demographic data (22 patients)

Variable	Value
Age (years, mean with SD)	65.4 ± 5.0
Female (cases, proportion)	17 (77.3%)
BMI (kg/m^2^, mean with SD)	26.8 ± 3.7
Follow-up time (months, median with IQR)	36.0 (31.0, 37.0)

*SD* standard deviation, *IQR* interquartile range, *BMI* body mass index, *OA* osteoarthritis

**Fig. 1:**
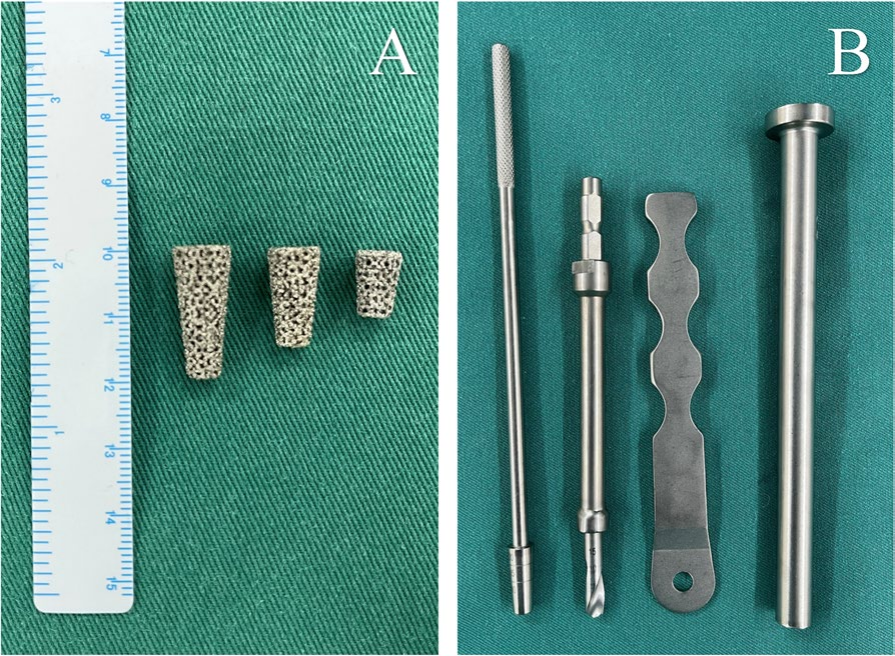
Porous metal pillar and instrumentation. **A** Porous metal pillars with different height. **B** Instrumentation comprised of drill, drill guide, trial, and impactor

The porous metal pillar (AK Medical Ltd., Beijing, China) is made of titanium and produced using 3D-printed technology with a porosity of 80%, pore size of 600–800 μm, and modulus of elasticity of 0.5–1.3 Gpa (Fig. 1). The pillar is implanted with a drill-based instrumentation. Six specifications are available with different dimension (6 or 8 mm) and height (10 mm, 15 mm, or 20 mm).

### Surgical technique

A midline incision with a medial parapatellar arthrotomy was used in all patients. After exposure, the osteophytes were removed. Bone cutting using measured resection technique and soft tissue balancing were performed in the usual fashion.

**Fig. 2:**
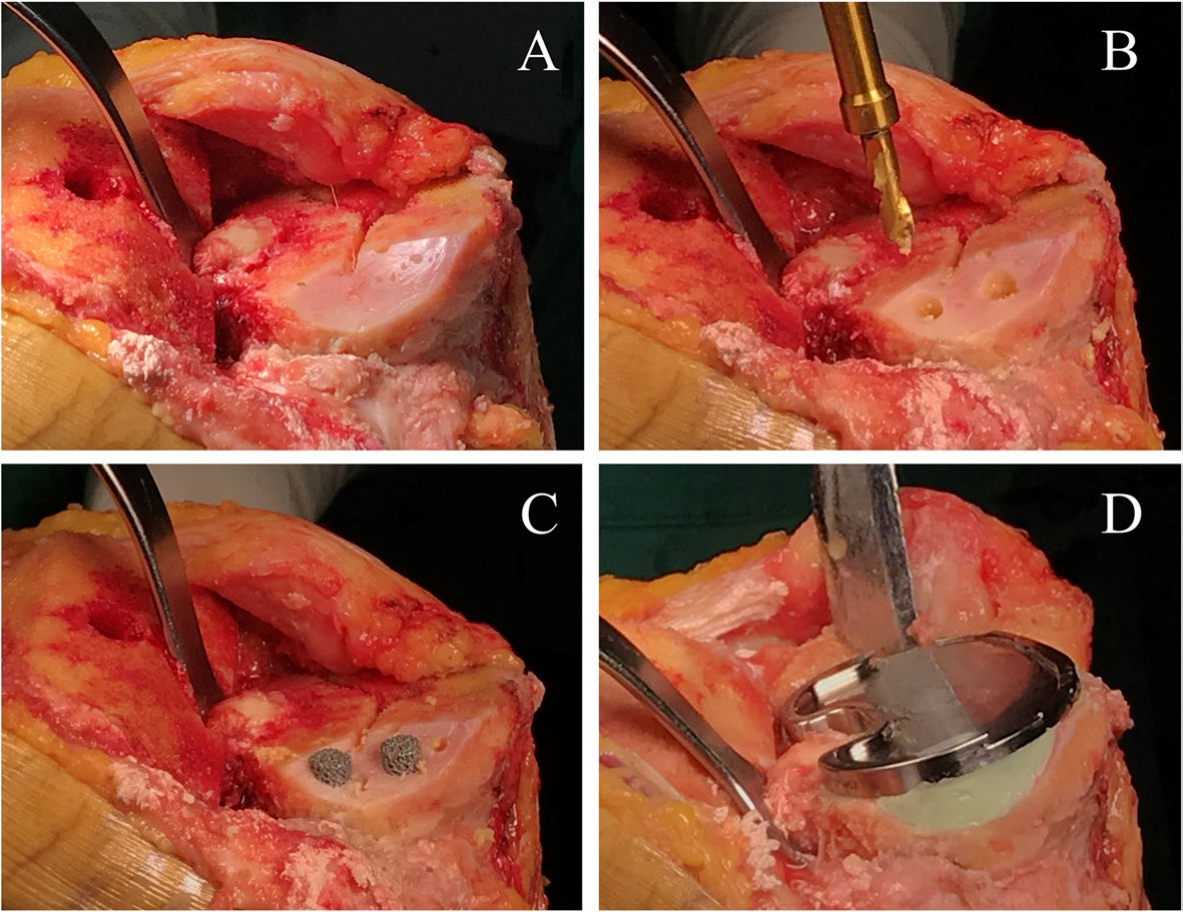
Porous metal pillar augmentation technique for uncontained tibial bone defect. **A** Bone defect was debrided. **B** Holes were drilled in the area of the bone defect. **C** The pillars were impacted into place with the top of the pillar just below the tibial cutting surface. **D** Bone cement was placed on the surface of the tibial resection, around the pillar, and in the area of the bone defect

The tibial bone was resected to a depth of 8 to 10 mm from the articular surface of the lateral tibial plateau in varus knee. Whereas in valgus knee, less bone should be cut initially from the lateral tibial plateau. The bone defects encountered in the present study were peripheral and uncontained, most in slop shape. Debridement of the bone defect was carefully performed (Fig. 2A). The depth of the bone defect was measured from the level of the tibial resection to the deepest point of the bone defect using a sterilized ruler. In general, metal pillar of 10 or 15 mm in height was chosen for bone defects of 5 to 10 mm in depth, and metal pillar of 15 or 20 mm in height was chosen for bone defects of 10 to 15 mm in depth. The number of metal pillars depended on the size of the bone defect. To implant a pillar into the area of bone defect, a hole was made using a drill (Fig. 2B). The diameter of the drill bit was in accordance with the pillar. The direction of drilling was perpendicular to the tibial cutting surface using a drill guide, and the depth of drilling was restricted by the drill guide with the aim of the top of the pillar being just below the tibial cutting surface. In general, the depth of the hole was between 5 and 10 mm. A pillar trial was inserted into the hole to check the direction, depth, and stability. Finally, the metal pillar was impacted into the hole using an impactor until the top of the pillar was just below the level of the tibial resection (Fig. 2C). Multiple small holes were drilled into the sclerotic area of the bone defect to facilitate bone cement penetration. A tibial extension stem was considered when there existed severe bone defect, poor bone quality, or significant soft-tissue imbalance. In this study, one case had severe tibial bone defect and compromised bone quality, Legion (Smith & Nephew, Memphis, TN, USA) was chosen with tibial extension stem to provide support for implant. Two cases had severe varus deformity and significant medial-lateral soft-tissue imbalance, LCCK (Zimmer, Warsaw, IN, USA) were chosen with constrained insert and extension stems to ensure knee stability. After the bone bed was thoroughly cleaned, bone cement was placed on the surface of the tibial resection, around the pillar, and in the area of the bone defect. The tibial component was impacted into place and the excess bone cement was removed (Fig. 2D).

All patients underwent a standard rehabilitation protocol similar to that for patients without bone defects. The day after surgery, patients were allowed to take full weight-bearing and start rehabilitation exercises.

### Clinical assessment

Patients were routinely followed up at 3, 6, 12 months, and annually thereafter. The knee and function scores of the Knee Society clinical rating system [13], the Western Ontario and McMaster Universities Osteoarthritis Index (WOMAC) [14], and range of motion (ROM) were assessed preoperatively and at each follow-up visit. Complications were identified from the medical records.

### Radiographic assessment

Standard plain films of the knee (anteroposterior, lateral, and merchant views) and long-leg standing radiographs were took preoperatively and at each follow-up visit. Femorotibial angle (FTA), the medial angle between the femoral and tibial anatomical axes [15], was measured on the anteroposterior radiograph preoperatively, postoperatively, and at the final follow-up. Hip-knee-ankle angle (HKAA), the medial angle between the femoral and tibial mechanical axes [15], was measured on long-leg standing radiograph preoperatively, postoperatively, and at the final follow-up. Radiolucent line and implant loosening were assessed according to the Knee Society roentgenographic evaluation system [16] on anteroposterior and lateral radiographs at each follow-up time point. Radiographic osseointegration was defined as absence of a lucent line between the host bone and the porous metal augment [17, 18] or presence of trabecular bone formation around the porous metal augment [19, 20].

### Statistical analysis

Continuous variables were expressed as means with ranges or medians with interquartile ranges (IQRs) according to the test of normality. Categorical variables were described as numbers and percentages. The preoperative and last follow-up clinical scores and radiographic parameters were compared using a paired-samples *t*-test or Wilcoxon signed-rank test based on the test of normality. SPSS 24.0 (IBM, Armonk, NY, USA) was used to perform statistical analyses. A *p*-value < 0.05 was regarded as statistically significant.

## Results

The tibial bone defects were peripheral and uncontained. The depth of the bone defects after tibial cutting was from 5 to 15 mm and the median was 7.0 mm (IQR: 6–9.5 mm). According to the classification of bone defects by Rand [1], there were 18 moderate bone defects and 6 extensive bone defects. Porous metal pillars of 10 mm in height were used in 9 knees, pillars of 15 mm in height were used in 11 knees, and pillars of 20 mm in height were used in 4 knees. One pillar was used in each of the 12 knees and two pillars were required in each of the other 12 knees.

The Knee Society scores and the WOMAC score improved significantly at the final follow-up assessment compared with the preoperative evaluation (Table 2). The median ROM increased significantly from 100.0° (IQR: 80.0–100.0°) preoperatively to 125.0° (IQR: 120.0–138.8°) at the last follow-up (*p* < 0.001).

**Table 2:** Clinical results of the patients (24 knees)

Variable	Preoperative value	Postoperative value	*P*-value
KSKS (median with IQR)	32.0 (25.0, 40.0)	94.0 (92.0, 95.0)	<0.001
KSFS (median with IQR)	50.0 (40.0, 57.5)	90.0 (90.0, 90.0)	<0.001
WOMAC (median with IQR)	50.5 (32.8, 58.5)	7.0 (7.0, 9.8)	<0.001

*IQR* Interquartile range, *KSKS* Knee Society knee score, *KSFS* Knee Society function score, *WOMAC* Western Ontario and McMaster Universities Osteoarthritis Index

**Fig. 3:**
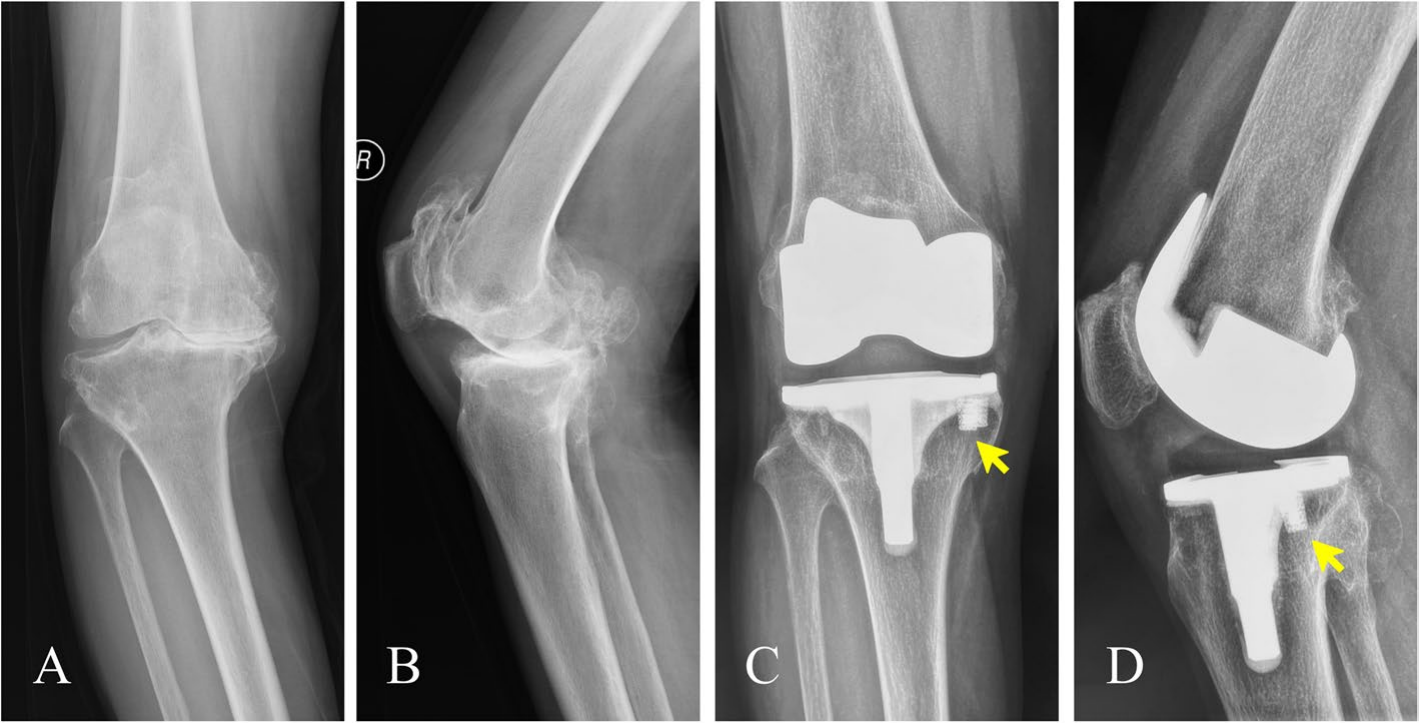
Preoperative and postoperative radiographs of a 64-year-old female patient. **A** Osteoarthritis was evidenced on the preoperative anteroposterior radiograph. **B** Osteoarthritis was evidenced on the preoperative lateral radiograph. **C** Trabecular bone formation (yellow arrow) around the pillar was noted on the anteroposterior radiograph 2.5 years after surgery. **D** Trabecular bone formation (yellow arrow) around the pillar was noted on the lateral radiograph 2.5 years after surgery

There were 22 varus knees and 2 valgus knees. The HKAA and FTA of the 22 varus knees were corrected after surgery (Table 3). There was no significant difference between postoperative HKAA and HKAA at the final follow-up (177.7 ± 1.7° versus 177.8 ± 1.6°, *p* = 0.556), and there was also no significant difference between postoperative FTA and FTA at the final follow-up (184.8 ± 1.1° versus 185.2 ± 1.6°, *p* = 0.066). For one valgus knee, the HKAA and FTA were corrected from 198.6° and 202.8° preoperatively to 183.7° and 187.4° at the final follow-up, respectively. And for another valgus knee, the HKAA and FTA were corrected from 204.7° and 208.2° preoperatively to 185.6° and 188.6° at the final follow-up, respectively. At the final follow-up, there was no evidence of loosening of any implant on the radiographs. Only one knee had a nonprogressive radiolucent line of 1 mm in width at the bone-cement interface in Zone one (edge of the medial tibial plateau) on the anteroposterior radiograph. No radiolucent lines were detected between the pillar and the adjacent bone in any of the cases. All pillars were considered stable with osseointegration, as evidenced by trabecular bone formation around the pillar and no radiolucent lines at the bone-pillar interface (Fig. 3).

**Table 3:** Radiographic results of the patients (22 varus knees)

Variable	Preoperative value	Postoperative value	*P*-value
HKAA (mean with SD)	163.9 ± 6.0	177.8 ± 1.6	<0.001
FTA (mean with SD)	170.8 ± 6.0	185.2 ± 1.6	<0.001

*SD* Standard deviation, *HKAA* Hip-knee-ankle angle, *FTA* Femorotibial angle

Complications such as neurovascular injury, fracture, or infection were not observed in the current study. There were no cases of prosthesis loosening, and none of the patients underwent revision surgery.

## Discussion

In this study, we reported the clinical and radiographic results of primary TKA with the use of novel porous metal pillars for tibial bone defects. The early clinical outcomes in terms of the Knee Society scores, the WOMAC score, and ROM were satisfactory, all pillars were considered stable with osseointegration, and there were no cases of implant failure at a minimum 2-year follow-up.

Traditionally, a variety of methods have been used for the management of tibial bone defects in primary TKA. However, the optimal treatment method for tibial bone defects has not yet been established. Ritter et al. [5] utilized bone cement with screws in 57 TKAs with tibial bone defects of 9 mm in mean depth and found no cases of component loosening after a minimum 3-year follow-up. However, a biomechanical study by Brooks et al. [6] demonstrated that bone cement filling provided inadequate support for the implant. In addition, radiolucent lines are frequently observed at the bone-cement interface [21, 22]. Autograft is an effective option to treat tibial bone defects [23, 24]. However, the amount of autogenous bone grafts obtained during primary TKA may not be sufficient. In contrast, allograft can be used for massive bone defects [25, 26]. In a study by Iwase et al. [26] using allograft for tibial bone defects ≥ 10 mm in depth, bone union was found in 94% of the knees at 96.4 months follow-up. Although the major advantage of bone grafting is restoration of bone stock, this technique has several complications such as grafted bone resorption, collapse, nonunion, fracture, and infection [7, 8]. Additionally, preparation of the grafted bone may be technically difficult and time-consuming. Metal augmentation is a useful method to address large bone loss [9, 10]. However, the use of metal augment needs further bone resection, which may reduce bone stock and make future revision surgery difficult. Furthermore, high incidence of radiolucent lines has been noted when metal augments are used [27–29].

Porous metal pillar augmentation is a new alternative method for bone defects encountered in knee surgery with the aim of providing mechanical support while achieving durable fixation. Due to the unique pillar geometry and metal material property, porous metal pillar can produce immediate and enduring structural support for the implant. The novel metal pillar is totally different from a screw used in bone cement filling technique, in which the role of the screw is to reinforce the bone cement. It is also unlike bone graft and doesn’t have the risk of grafted bone cracking, resorption, and collapse [7, 8]. For most treatment methods for tibial bone defects, immediate full weight-bearing can be given postoperatively [26, 29]. However, Watanabe et al. [30] evaluated 30 knees with autologous bone grafting for tibial defects in TKA. The graft was fitted into place without screw fixation. In their study, partial weight-bearing was allowed 3 weeks after surgery and full weight-bearing was allowed 6 weeks after surgery. In the present study, patients were allowed immediate full weight-bearing and early rehabilitation exercises after surgery. All patients had improved Knee Society scores, WOMAC score, and increased ROM. The varus or valgus deformities were corrected. There were no cases of implant loosening. Only one knee had a nonprogressive radiolucent line at the bone-cement interface. In contrast, the incidences of radiolucent lines are much higher when bone cement and traditional metal augments are used. Lotke et al. [22], in a series of 59 knees with the use of bone cement for tibial bone defects, observed radiolucent lines in 43 (77%) of the knees. Tsukada et al. [29] reviewed the clinical results of 33 TKAs with metal blocks for tibial bone defects, and reported that the incidence of radiolucent lines was 30.3%.

This porous metal pillar has rough surface and high porosity structure. The primary press-fit stability of the pillar is achieved after the pillar is impacted into the drilled hole. High porosity structure of the pillar has the potential to promote bone ingrowth and provide long-term biologic fixation. Traditionally, porous metal cones have been used to treat massive bone defects in revision TKA and have gained promising results. Erivan et al. [11] reviewed 61 patients who undergone revision TKA using porous tantalum cones. The 5-year survivorship with tibial cone revision for aseptic loosening was 100% and 93.4% for all-cause revision. Tetreault et al. [12] analyzed 142 revision TKAs using porous titanium metaphyseal cones with the mean follow-up time of 2.4 years. Their study showed 100% of survivorship free of cone revision for aseptic loosening and 98% of survivorship free of any cone revision. The novel porous meta pillar is made of titanium and produced using 3D-printed technology with a porosity of 80%. The osseointegration around the porous metal pillar was favorable in our study, and no radiolucent lines at the bone-pillar interface were detected in any of the cases. All the pillars were stable without loosening and the survivorship was 100% at a minimum of 2-year follow-up.

A major advantage of this porous metal pillar is the simplified surgical technique. The drilling process with a drill guide is quick and precise. The metal pillar is then impacted into place using an impactor. The surgical efficiency of this new technique is superior to bone grafting and traditional metal augmentation. When bone grafting is used for tibial bone defects, it is technically difficult to shape the grafted bone to match the defect, and the surgical times would be extended [24, 26]. While using traditional metal augment, the deficient area needs to be prepared in slope or rectangular shape to fit the metal wedge or block. This is also time-consuming and leads to further bone loss. In contrast, although bone removal by drilling is needed for putting the pillar, the amount of bone loss is relatively small. In addition, the porous metal pillar has the advantage of extensive modularity. The use of pillar doesn’t interfere with the tibial tray and tibial keel. Moreover, different specifications are available and several pillars can be used in combination, which makes this technique applicable to various bone defects. In this study, the moderate and extensive tibial bone defects were well managed with the use of porous metal pillars.

This study has several limitations. First, this study has a relatively small number of cases with a short-term follow-up time period. The reason is that the application of this novel porous metal pillar in our hospital is not so long. Second, the current study has not been designed as a comparative study, and we can’t directly compare this new technique with other methods. However, the results of this study are encouraging and may have an important impact on the clinical practice. Third, the severity of the tibial bone defects was variable and heterogeneous in the present study. However, the moderate and extensive bone defects were both well managed with the use of porous metal pillars, which verified the effectiveness of this new technique for various bone defects. Finally, the use of pillar adds additional cost. Because this is a small case series study with the aim to report the short-term clinical and radiographic results of porous metal pillars, we did not perform the cost-benefit analysis. Cost-benefit study can be conducted in the future with more related information.

## Conclusions

The use of novel porous metal pillars yielded satisfactory clinical outcomes and reliable radiological evidence of fixation in this study with a minimum 2-year follow-up. Porous metal pillar augmentation can be considered as a valuable and easy-to-use method for the management of tibial bone defects in primary TKA. Future studies with larger populations and longer follow-up are needed to determine the long-term clinical success of this new technique.

## Data Availability

The datasets used and /or analyzed during the current study are available from the corresponding author on reasonable request with the permission of the Institutional Review Board.

## Abbreviations

TKA: Total knee arthroplasty
BMI: body mass index
SD: standard deviation
IQR: interquartile range
OA: osteoarthritis
KSKS: Knee Society knee score
KSFS: Knee Society function score
WOMAC: Western Ontario and McMaster Universities Osteoarthritis Index
HKAA: hip-knee-ankle angle
FTA: femorotibial angle
RL: Radiolucent line
